# ‘*Trial and error…’, ‘…happy patients’ and ‘…an old toy in the cupboard’*: a qualitative investigation of factors that influence practitioners in their prescription of foot orthoses

**DOI:** 10.1186/s13047-016-0142-9

**Published:** 2016-03-22

**Authors:** Anita Ellen Williams, Ana Martinez-Santos, Jane McAdam, Christopher James Nester

**Affiliations:** School of Health Science, University of Salford, Brian Blatchford Building, Salford, M6 6PU UK

**Keywords:** Foot orthoses, Technology, Qualitative research, Focus groups

## Abstract

**Background:**

Foot orthoses are used to manage of a plethora of lower limb conditions. However, whilst the theoretical foundations might be relatively consistent, actual practices and therefore the experience of patients is likely to be less so. The factors that affect the prescription decisions that practitioners make about individual patients is unknown and hence the way in which clinical experience interacts with knowledge from training is not understood. Further, other influences on orthotic practice may include the adoption (or not) of technology. Hence the aim of this study was to explore, for the first time, the influences on orthotic practice.

**Methods:**

A qualitative approach was adopted utilising two focus groups (16 consenting participants in total; 15 podiatrists and 1 orthotist) in order to collect the data. An opening question *“What factors influence your orthotic practice?”* was followed with trigger questions, which were used to maintain focus. The dialogue was recorded digitally, transcribed verbatim and a thematic framework was used to analyse the data.

**Results:**

There were five themes: (i) influences on current practice, (ii) components of current practice, (iii) barriers to technology being used in clinical practice, (iv) how technology could enhance foot orthoses prescription and measurement of outcomes, and (v) how technology could provide information for practitioners and patients. A final global theme was agreed by the researchers and the participants: ‘*Current orthotic practice is variable and does not embrace technology as it is perceived as being not fit for purpose in the clinical environment. However, practitioners do have a desire for technology that is usable and enhances patient focussed assessment, the interventions, the clinical outcomes and the patient’s engagement throughout these processes’.*

**Conclusions:**

In relation to prescribing foot orthoses, practice varies considerably due to multiple influences. Measurement of outcomes from orthotic practice is a priority but there are no current norms for achieving this. There have been attempts by practitioners to integrate technology into their practice, but with largely negative experiences. The process of technology development needs to improve and have a more practice, rather than technology focus.

## Background

Foot orthoses are used to manage a plethora of foot and lower limb conditions, including those associated with diabetes [1] and rheumatoid arthritis [2]. They are advocated in several practice guidelines [3–7] with the standards being defined by the emerging evidence base. However, these guidelines fall short of detailing precisely the type of orthosis, how to derive its shape or what materials should be used. Therefore, what an individual practitioner chooses to provide for each patient may be based on personal preference and hence may result in variable practice.

In relation to underpinning theory and protocols to assess patient suitability for orthoses, we assume practitioners follow the widely held Root model of orthotic practice [8, 9] since this was likely to be the foundation for their initial training. However, Jarvis et al. [10] found evidence that practitioners adapt elements of the model as they gain experience and decide for themselves which parts of the assessment protocols are most valuable. Alternative approaches to foot assessment, such as the Foot Posture Index [11] might also inform clinical decisions related to orthosis prescription. Whilst these and other approaches to patient assessment might provide a general approach to orthotic prescription, the final choice to use a customised or prefabricated orthosis, the choice of orthotic material, and nature and scope of advice provided to patients, lies with individual practitioners. These decisions may also be affected by factors specific to the patient (e.g. footwear choices, expected time scale, prior experiences of orthotic use) or practice context (e.g. cost of orthoses and clinical appointments, time available). Freedom to decide the details of the orthotic prescription allows a suitable level of autonomy that places the requirements of the patient first. Equally, however, it might allow such freedom that standardisation of best practice is difficult to achieve. Whilst the theoretical foundations for the use of foot orthoses might be relatively consistent, actual practices and therefore the experience of patients is likely to be less so. Furthermore, the factors that affect the decisions that individual practitioners make about individual patients have not been explored. Thus, the way in which clinical experience and local constraints interact with knowledge from formative training in orthotic practice is not understood.

Other factors that should influence orthotic practice include the policies that encourage greater adoption of technology in practice and the generally improved availability of technologies. In terms of foot assessment for example, 3D foot scanning has been shown to more reliable than plaster of Paris and foam impression boxes [12]. However, whilst not a new technology, anecdotally, it does not seem to have found favour in the majority of health care settings. Also, the drive for ever more quantitative evidence of outcomes from practice should mean that there will be a role for measurement tools that quantify changes due to foot orthoses, such as a change in plantar pressure. However, in shoe pressure measurement tools appear limited to the research rather than clinical domain, and clinical outcome measures seem routed to more subjective tools such as the Manchester Foot Pain and Disability Index [13] possibly due to ease of administration and being less time consuming. Thus, there is a disconnection between health technology policy, what technology can do to enhance practice and the adoption of the apparently useful technology.

Despite foot orthoses being one of the main interventions for foot pathology [14], there is a lack of knowledge about current orthotic practice and the use of technology within it. To date, there has been little published work investigating prescribing practices, with the focus being describing practice trends [15] rather than explaining the factors that influence decisions. This is the first study that has the primary aim of gaining insight into what constitutes current foot orthotic practice, the factors that affect this area of practice and how technology has or may play some role in future orthotic practice. The purpose of this work was to better understand the context within which future innovations in practice might be introduced. Accordingly, this study set out to explore such issues as, alternative methods for assessing patients and their feet (e.g. 3D and 4D scanning), use of new orthotic materials (e.g. additive manufacturing), and adoption of technologies that might enhance clinical processes, patient experiences and evaluation of outcomes (e.g. mobile and web technologies).

## Method

This study adopted a qualitative approach utilising two focus groups in order to collect the data and a thematic framework to analyse the data. Following ethical approval from the University of Salford ethics committee (HSCR12/62) the participants were purposively recruited as being experts in the assessment of patients with lower limb pathology requiring foot orthoses. Podiatrists were identified from the North West Clinical Effectiveness groups (Diabetes, Rheumatology and the Extended Scope Practitioner groups) and orthotists were invited to reflect the other main profession involved in orthotic provision in the UK. The inclusion criteria were that they provided foot orthoses within their practice and had more than two year’s clinical experience. Information was provided to the group members by email about the proposed study. Those who expressed an interest (*n* = 17) received a Participant Information Sheet, 4 weeks before the study to allow participants to consider their involvement in the study. They all agreed to take part in the study and provided informed and written consent.

There were two focus groups of eight participants in each (one participant did not attend on the day they were allocated to). All were podiatrists except one who was an orthotist. The focus groups were facilitated by AW, an academic who has experience of running focus groups and previous experience of clinical practice in this area, supported by AM and CN who took field notes. The focus groups were recorded using a digital data recorder. Following introductions and an overview of the purpose of the focus group, the opening question was asked followed by trigger questions to guide the conversation (Table 1).

**Table 1:** Focus group questions

Opening question	*“What factors influence your orthotic practice?”*
Examples of trigger questions	*What influences the design of the foot orthoses provided?*
	*What factors influence how you assess patients?*
	*How do you evaluate whether the foot orthoses are successful in meeting the clinical and patient focussed outcomes?*
	*How do you think the technological advancements might improve your practice and the outcomes from foot orthoses and footwear?*

The data generated from the dialogue was transcribed verbatim and analysed using thematic analysis [16]. This process of analysis involves the authors independently identifying recurring statements and meanings. These were agreed on and then organised into subthemes, themes and a final ‘global’ theme as an overall conclusion.

Following analysis, the results were sent by email to the focus group participants for verification and additional comment. There were no additional comments or additions. This adds to the truthfulness of the results. Each participants name was replaced with a pseudonym in order to maintain anonymity.

## Results

All the participants were NHS employed (four were Extended Scope Podiatrists) and all specialists in orthotic provision (mix of musculoskeletal [paediatric and adult] and diabetes). There was also one orthotist who managed most patient groups. Most had been qualified for more than 5 years, with 8 of them being qualified for more than 10 years.

### Theme 1: influences on current practice

There were four subthemes to Theme 1: the influence of participants’ undergraduate training, clinical experience, the influence of the service they work in, and evidence from research. How the participants were *trained* as undergraduates influenced their decisions in relation to patient assessment, orthotic prescription (design and materials). As Peter reveals,

*“…I trained at XXX but my colleague trained at XX and we do differ in our approach and choice of materials in particular…”*

Further to basic training, *clinical experience* of what works also influences practice as Sophie said,

*“…once you have been trained then it’s a matter of trial and error…what works gets repeated and what doesn’t…well you bin that idea. Then all this becomes your personal preference.”*

In relation to the influence of the *type of service* they worked in, some were working within podiatry teams with their peers, whilst some were working within wider multidisciplinary teams where, as Lesley pointed out,

*“…you have to retain your professional identity and so this leads to behaviours becoming entrenched rather than changing to keep up with new practice…”*

Lack of time in the consultation was an issue that influenced the participants’ decisions (often this was under 20 mins for assessment and diagnosis) and this was independent of the service type. However, many provided chairside foot orthoses at the first appointment so that a second appointment was not required or if they wanted to ‘test’ whether an orthosis was going to help before ‘investing’ in a bespoke device. As Tom revealed,

*“…this may sound expensive and time consuming…at least we can give something on the day or if you are unsure of whether an orthotic is going to work then you can give it a go with the temporary one.”*

It was also revealed that compared to those based in podiatry services, those in the Independent Care Assessment and Treatment service had no opportunity for reviewing the patient. This means that these clinicians have no idea if their interventions have worked or not. Stephanie identified this as a big problem,

*“…you have to assume that if they don’t come back then it has worked…I fear that many don’t though. Some do get referred back in again as another ‘episode’ but time has dragged on and the condition has often deteriorated…”*

All of the participants agreed that most of their work involved treating problems when they had become chronic, rather than in prevention. This reflected their workload being with high risk groups rather than low risk. Evidence from research was considered as the least influence (unless it had been part of their training). However, when it did, they adapted the evidence to their own practice, with time and available materials being the main constraints to applying evidence. Also, as Andrea stated,

*“…we need to feel that there is a sense of ownership rather than being told what to do by researchers…when I read papers I have to apply it to what I know works and often that is in conflict…I then dismiss the research.”*

All agreed that a consensus opinion from clinical experts should be merged with research evidence to ensure that translation into practice would be both useful and effective.

### Theme 2: components of current practice

There were three subthemes to Theme 2 that reflect: the components of current practice, the consultation, the intervention and the outcome. The most important one was seen as a patient focussed consultation, as Jade revealed,

*“Listening to the patient and history taking is a huge part of the consultation (time)…it is part of getting to the correct diagnosis and patients expect it and I see it as the foundation of success…then the ‘hands on’ bit has to be quick so I tend to use foam boxes for casting or off the shelf foot orthoses.”* Others agreed with this and Sam added,

*“…the consultation is where you can educate the patient and that is as important, if not more so, than the orthotic…if they understand then they will change their footwear and then that’s half the battle.”*

The consultation is seen as including history taking and assessment in order to achieve a diagnosis. However, it is also considered an opportunity for achieving both positive patient engagement with appropriate health behaviours in order to achieve maximum health benefits.

Further, Andrew describes this as an opportunity achieving the balance between the patients’ aims and the clinicians,

*“…we listen to what patients want and then make that fit with what we want. I spend a lot of time engaging with the patient…counselling them on the effect of weight and things like their type of activity…”*

The second component is the *intervention,* which may require tailoring to the individual. As Ciaran revealed,

*“…I often compromise…. Don’t always do a full correction…an example is the height of the arch as it may irritate, shoe choice may not be suitable so full correction isn’t possible.”*

Despite this compromise and the challenge of the patient’s choice of footwear, the foot orthosis design is based on the foot type, foot condition and the aim of treatment, as David said,

*“…we may be aiming for pressure redistribution, improve function, reduce shock and shear or combinations of all of these…this defines what type of device and the materials.”*

Further to this, the majority agreed that they worked on the principle of, *“…the least intervention for the maximum effect”* with the *“…all singing and dancing versions for when these don’t work”.*

The third component was measurement of outcomes. Despite being seen as important, quantifiable outcomes were generally not measured, with *“…happy patients”* being reported to be the best indicator of success. After debate, it was concluded that reduction in pain was the best ‘metric’ for most patients, with a visual analogue scale being both quick and easy to administer. Generally, the participants were not focussed on using quantifiable measures or tools. This was due to time constraints or there being no review system in place in order to compare the baseline measurement with the outcome.

### Theme 3: barriers to technology being used in clinical practice

All the participants reported that they did not use technology, such as foot pressure assessment. Five reported that their service had invested in pressure measurement systems but there were barriers to its use and hence, *“…it’s just sat like an old toy in a cupboard*”. The barriers to the use of technology were in three main sub-themes; its usability, lack of training in its use, and its perceived ‘value’ in the diagnosis and monitoring process. The main issue with usability was health and safety, with cables meaning that patients may trip and fall. Additionally, as Simon reported, the information produced by these systems was difficult to interpret,

*“…it provided too much info…its ok for research but for clinical use it is difficult to navigate through all of it…you normally use 10 % of the software, because most of the information is not useful for clinic, it is for research…it also doesn’t replicate the foot in sufficient detail. The manufacturers don’t produce kit that is clinically useful”.*

Also, the terminology was perceived to be too technical and complex, as was the setting up of the system. As Tracey reported, *“…it needs setting up differently for different clinicians…this takes too much time…and it needs to be calibrated”.*

In relation to being *trained* in the use of these systems, most agreed with Simon in that,

*“We are not trained in technology…we would spend too much time to set up and to interpret, I don’t think patients expect it…this makes it slow to use and maybe wrong information will be collected.”*

There was a strong sense that the consultation, diagnosis and prescription should not be done *“…at the touch of a button”.* This indicates their desire to retain ownership of the process by the clinician and to maintain their responsibility for decision making.

### Theme 4: how technology could enhance foot orthoses prescription and measurement of outcomes

Despite the reservations expressed in Theme 3, there was a clear voice about what technology could do if the clinician’s needs were listened to and addressed. There were four subthemes within Theme 4, including; footwear suitability, taking foot impressions, the design of foot orthoses, and evaluation of actual and/or potential outcomes.

Footwear was seen as the biggest obstacle to the success of the foot orthoses and hence, as Sarah summarises,

*“Shoes have to be an essential component…we need an algorithm for orthoses AND shoes in the context of the patients life…also something to inform the patient what a good shoe is and then the orthoses would go into retail footwear based on foot dimensions and volume.”*

Further, as Fred suggests, technology could,

*“…evaluate the dimensions and design of existing footwear to check suitability and if the prescription changes in relation to the shoes chosen…for example trainers often have a ‘posted’ element.”*

In relation to reducing the need for taking foot impressions and improving the design of foot orthoses, Sophie suggested the use of *“…templates so you just have to introduce foot measures without casting, and that the software tells you the best design and material”.* In addition to this, Peter suggested a library of shapes (overall design and additions) but *“… not too many as it would get too complicated to navigate through in the time we have”.* Many agreed about the potential for enhancing the design of foot orthoses, as Andrea suggests,

“…being able to evaluate modifications or features of a design…. Such as the exact replication of the contours of the plantar surface of the foot for total contact foot orthoses.”

Interestingly, although most do not measure outcomes, they all saw the potential for incorporating this within technology. As Sally articulates,

*“…to be able to assess how the foot orthoses are working before the patient leaves the clinic…in order to make adjustments that would normally be done at the review when problems might have occurred…could predict this…and very useful when you don’t have a review appointment.”*

However, Neil expressed caution here,

*“….you can only predict the effect of the orthoses, not the success, as there are too many extrinsic factors that influence this…you can have the same foot type, but if you put that in two different patients there is a chance that you will get two completely different responses by doing exactly the same thing”.*

Nevertheless, most agreed it would be a useful way to capture outcomes such as pain reduction, although some thought that this alone may not tell the whole picture. Levels of activity were suggested as a better outcome to measure. However, they also recognised that additional factors such as medication may contribute to successful outcomes and hence needed to be documented.

### Theme 5: how technology could provide information for practitioners and patients

The focus of this theme was the provision of information for both practitioners and the patients. Some suggestions for patient education were made. Frank suggested that,

*“…If we could check if the insole is working inside the shoe, and that way be able to show the patient the treatment is working correctly…it may increase compliance.”*

Aligned with this Lesley suggested,

*“A visual for what is a good shoe (components identified and then jigsaw together as a whole picture…patients see shoes as a whole unit not the component parts and so this ‘deconstruction’ would be a useful visual aid.”*

For the purpose of supporting the continued education of practitioners, it was agreed that it would be useful to have the interpretation and translation of research into practice. However, Sally also highlighted the need for clinicians to remain autonomous and suggested that, *“…what we need is synthesis of research information to inform decisions, not instruct what we should and shouldn’t do”.*

Two of the main benefits of technology were the collection of data on outcomes (to defend practice and services) and standardisation of practice. As Sophie suggests, *“…sometimes patients get conflicting views and interventions”.*

From these five themes, a global theme was agreed between the authors and verified with the participants. The global theme summarises the results in that,

*Current orthotic practice is variable and does not embrace technology as it is perceived as being not fit for purpose in the clinical environment. However, practitioners do have a desire for technology that is usable and enhances patient focussed assessment, the interventions, the clinical outcomes and the patient’s engagement throughout these processes.*

## Discussion

The aim of this work was to gain insight into what constitutes current foot orthotic practice. Further, we aimed to identify, for the first time, the factors that affect this area of practice and how technology does or may have a role to play. This study has demonstrated that for these practitioners, there is no singular algorithm for the prescription of foot orthoses. Current practice varies and is borne of a complex interaction between formal education and local influencing factors. Whilst these practitioners experienced largely common teaching in their initial undergraduate training, other subsequent factors influence how this training is executed in actual practice.

Orthotic prescription choices tend to be based on the practitioners’ preferences, with ‘trial and error’ being the foundation for how clinical experience is blended with a formal understanding of foot structure, biomechanics and orthotic principles. The variation in practice is not necessarily a problem, as it reflects the real influences that need to be taken into account to offer patient specific care and within the framework of the services in which care in delivered. This variation may also be acceptable if the scope of practice is different (e.g. musculoskeletal vs high risk foot care), with the nature of the biomechanical and clinical objectives often overlapping (e.g. pressure relief, change in foot motion). However, the local factors influencing practice are not evident in any theoretical model of orthotic practice and hence this may leave contemporary educational materials and research evidence too remote from the reality of practice.

Through their clinical experience and blending of various influences, the focus for clinical practice appears to have shifted away from achieving a defined biomechanical objective, towards what delivering what patients want. This was evident through their focus on the patients’ needs within the focus group discussions. In relation to the expected outcome of foot orthoses, this was reinforced by the patient specific notion of “success”, rather than biomechanical “correction” as defined by the theoretical models of orthotic practice. Recognition that outcomes should be patient, rather than practitioner focussed, is evidence that the application of the Root paradigm in orthotic practice [8, 9] has been influenced by contemporary health policy in the United Kingdom [17].

There was also evidence of orthotic practice having evolved due to changes in professional roles, and thus orthotic practice moving beyond the provision of a “biomechanical device”. Participants referred to “counselling” of patients, and consideration of wider issues such as levels of activity and weight management as being interrelated factors that affect foot or lower limb health. These practitioners are providing care that blends mechanical intervention with appropriate health behaviour and self-management strategies. This is an important outcome of this study in terms of understanding their professional roles.

Practitioner knowledge of the success or failure of foot orthoses seems to be determined largely by patient behaviour and whether they return to the service or not. Measurement of success is rarely captured using validated patient-reported outcome measures, with the current focus being on “happy patients” or levels of satisfaction [18]. This is underpinned by the implied assumption that a ‘non-returning’ patient is a marker for a good outcome. It is quite possible that for a proportion of the patients concerned, ‘non- returning’ indicates a change in circumstances (such as in occupation), rather than a health improvement directly associated with the intervention. At best, this provides outcome data for service managers, such as the demand for appointments. At worst it grossly overestimates the effectiveness of the orthotic service. Data on patient outcomes is an area devoid of quality and coherent approaches, and in urgent need of attention.

Research outcomes, as embedded in clinical guidelines, are widely seen as the bedrock of evidence based practice [19]. However, based on the work presented here, the influence of guidelines on orthotic practice seems limited. The problem from the participants perspective was a difficulty in translating research into real world practice. This points to the need for greater involvement of clinicians within the research communities in the planning and implementation of research in order to create the evidence that is relevant to them. This issue may also reflect the state of orthotic research, in that fundamental knowledge may not yet be sufficient to support the design of high quality ‘practice relevant’ research. At this point in time, the evolution of research in this area may reflect the availability of research resources, which perhaps favour ‘laboratory based’ research over ‘practice relevant’ research.

The participants believed that technology should and could enhance practice. However, prior negative experiences with technology mean that even with access to a preferred technology, it may not get used. It was recognised that technology has the potential to enhance all aspects of orthotic practice, from assessment of the foot, education of the patient, through to measurement of outcomes. If such information was gathered routinely and on a systematic scale, a wider and quantitative picture of foot orthosis prescription practices and outcomes of services could emerge. This might prove invaluable to innovate and support the maintenance of services.

However, technology needs to add value to practice without adding to the burden of work. There was a shared experience that technologies had become *“…an old toy in the cupboard”*, a seemingly common fate of foot and orthotic-related technologies. There is therefore a need for technology to be designed to meet the practical needs of clinicians. The developers of technology need to gain a better knowledge of the realities of practice earlier in the development processes, in order to ensure the transition from the manufacturers’ ‘benchtop’ to clinical practice. This challenge may reflect the fact that in some cases the technology was developed initially for research. This, we assume, was followed by promotion of the technology to clinical settings, without sufficient tailoring of the technology to the operational realities of practice. Making clinical application of a technology an explicit objective of technology development may improve this issue.

Integrating technologies with consumer products may be an alternative strategy and remove some practical barriers (e.g. apps for mobile phone and tablet platforms). There are already examples of good practice where complex technologies can be developed in a way that makes clinical use fast and easy. Ultrasound, for example, is a very complex medical physics imaging tool, yet the use of the device is both fast and routine in many clinical settings.

There was evidence of some concern from the participants as to the impact of technology in respect of their role. Clearly, in enhancing clinical processes there is the potential for tasks that are currently undertaken by clinicians to be replaced by technology. However, given the variety in practice identified, it seems unlikely that technology could ever automate such complex and nuanced decision making on a patient by patient basis. Rather, technology can support decision making and autonomy by adding value and assisting in their role. Whilst not discussed within the focus group, the variety in decision making might be far less in some areas than others and therefore, some decisions could be automated or standardised. However, the added value here could be to leave practitioners greater time to focus on more complex cases, where their ability to undertake complex assessments and decision making is better utilised.

An important potential limitation of our work is that preselected participants were employed in health services in the United Kingdom, and this could affect the generalisability of the outcomes to other professions and other health care settings. However, we invited experienced practitioners who drew on their knowledge of other practitioners within their own services and networks. Further, the purpose of qualitative studies such as this, is to gain deeper knowledge and insight from a select group of participants who have particular knowledge and experience of a ‘phenomenon’ [20], in this case, the prescription and provision of foot orthoses. Indeed, one of the aims of this study was to reveal the nature of current practice and given the variation in practice and influencing factors involved, a wider exercise seems pertinent. This might quantify the variations in nature and scale of orthotic practice and allow associations to specific issues to be investigated. There is a lack of information about the provision of foot orthoses, including how it may vary between all the professional groups involved. Hence, further investigation of the nature, scale and variation in orthotic practice is warranted.

## Conclusion

The practice of providing foot orthoses varies considerably between practitioners reflecting the integration of formal education with local factors influencing their personal practice style. The influence of research on practice was less evident. Measurement of outcomes from orthotic practice is a priority but there are no current norms for achieving this. There have been attempts by practitioners to integrate technology into their practice, but with largely negative experiences. The process of technology development needs to improve and have a more practice rather than technology focus. Further, more information on current orthotic practice is needed.
